# The association between anthropometric measures and glycated haemoglobin (HbA1c) is different in Russian, Somali and Kurdish origin migrants compared with the general population in Finland: a cross-sectional population-based study

**DOI:** 10.1186/s12889-019-6698-0

**Published:** 2019-04-11

**Authors:** Natalia Skogberg, Tiina Laatikainen, Eero Lilja, Annamari Lundqvist, Tommi Härkänen, Päivikki Koponen

**Affiliations:** 1 Department of Welfare, National Institute for Health and Welfare, Mannerheimintie 166, PL 30, 00271 Helsinki, Finland; 2 Department of Public Health Solutions, National Institute for Health and Welfare, Mannerheimintie 166, PL 30, 00271 Helsinki, Finland; 3 Institute of Public Health and Clinical Nutrition, University of Eastern Finland, PL 1627, 70211 Kuopio, Finland; 4 Joint municipal authority for North Karelia social and health services, Tikkamäentie 16, 80210 Joensuu, Finland

**Keywords:** HbA1c, Glucose, Ethnicity, Migrant, BMI, Waist circumference, Waist-to-height ratio, Russian, Somali, Kurdish

## Abstract

**Background:**

Persons of African and Middle-Eastern origin living in European countries have a high prevalence of type 2 diabetes, accompanied by high prevalence of obesity among women but not always among men. The aim of this study was to examine whether there are differences in the association between anthropometric measures and glucose levels measured with glycated haemoglobin and fasting blood glucose among persons of migrant origin in Finland.

**Methods:**

Cross-sectional population-based data of the 30–64 year-old participants in the health examination of the Migrant Health and Wellbeing Study was used, selecting persons without diabetes (Russian origin *n* = 293, Somali origin *n* = 184, Kurdish origin *n* = 275). The reference group were non-diabetic participants in the Health 2011 Survey (*n* = 653), representative of the general Finnish population. Anthropometric measures included body mass index (BMI), waist circumference (WC), waist-to-height ratio (WHtR) and waist-to-hip ratio (WHR, available for Maamu Study participants only).

**Results:**

Depending on whether continuous or categorical anthropometric measures were used, age, sex and anthropometrics explained 13–18% of variation in HbA1c among persons of Russian origin, 5–10% among persons of Somali origin, 1–3% among persons of Kurdish origin and 11–13% among the general population. Also depending on whether continuous or categorical anthropometric measures were used, age, sex and anthropometrics explained 13–19% of variation in fasting blood glucose among persons of Russian origin, 15–20% among persons of Somali origin, 13–17% among persons of Kurdish origin and 16–17% among the general population. With exception for BMI, strength of the association between continuous anthropometric measures and HbA1c was significantly lower among persons of Kurdish origin compared with the general Finnish population (*p* = 0.044 for WC and *p* = 0.040 for WHtR).

**Conclusions:**

A low degree of association between anthropometric measures and HbA1c was observed among persons of Kurdish origin. Findings of this study suggest caution is warranted when using HbA1c as a screening tool for glucose impairment among persons without diabetes in populations of diverse origin.

**Electronic supplementary material:**

The online version of this article (10.1186/s12889-019-6698-0) contains supplementary material, which is available to authorized users.

## Background

According to 2015 estimates, 415 million persons have been diagnosed with type 2 diabetes (T2D) and a further 193 million persons were unaware of having the condition [1]. Prevention and early detection of persons at increased risk for T2D plays a central role in counteracting the growing burden of disease [2]. It is well established that obesity, particularly abdominal obesity, is a central precursor to T2D [3]. Increase in weight is proportionally associated with increase in glucose levels and hence increased risk for T2D [4]. Glucose levels are measured with fasting blood glucose, 2-h blood glucose levels following an oral glucose tolerance test or glycated haemoglobin (HbA1c) [5]. While there is a general consensus that blood glucose and HbA1c may be used interchangeably for detecting T2D, use of HbA1c for screening of pre-diabetes is disputed. The American Diabetes Association (ADA) recommends the use of HbA1c in pre-diabetes screening [6], whereas the World Health Organisation (WHO) calls for caution due to scarcity and inconclusiveness of evidence on the degree of correlation between fasting blood glucose and HbA1c [5]. In particular, there is a scarcity of previous research on the correlation between fasting blood glucose and HbA1c among persons of Middle-Eastern and African origin without diabetes [7, 8]. In previous studies, fasting blood glucose and HbA1c had a relatively good degree of correlation in the Iranian population [9], whereas a low degree of correlation was found among persons of African origin [8].

It is estimated that 65–80% of T2D cases can be prevented through a reduction of overweight and obesity [10]. For this reason, measurements of general obesity with BMI and abdominal obesity with WC are central components of non-invasive T2D risk assessment tools commonly used in clinical practice [11]. In addition to BMI and WC, obesity can also be measured with other anthropometric measures, including waist-to-height ratio (WHtR) and waist-to-hip ratio (WHR) [4, 12]. The advantage of WHtR is that it reflects accumulation of surplus fat in the abdomen area as well as intra-individual differences in height [13, 14]. Waist-to-hip ratio (WHR) has been used to a lesser extent as it is mainly considered to be an indicator of body shape rather than of accumulation of fat in the abdomen area [4].

Majority of studies on the association between obesity and T2D have been conducted among predominantly White populations in high-income countries [11, 15]. With increasing population diversity, this association has been examined also among populations of migrant origin [16]. A number of studies have reported a lower degree of association between obesity measured with BMI and WC and T2D among persons of South Asian, Middle-Eastern and African origin [17–19]. Studies examining the association between other anthropometric measures with T2D among persons of migrant origin are scarce [16]. Proposed explanations for the reported lower degree of association between BMI and WC with T2D among persons of migrant origin include differences in biological mechanisms [20], family history [21, 22], epigenetic factors [23] and lack of established cut-offs for obesity among persons of African and Middle-Eastern origin [24–26].

Differences in the exposures over the life course among migrating populations as well as the effect of migration process in itself may alter biological pathways to disease, leading to varying disease profiles among migrating populations both in comparison with populations in the country of origin and the general population in the country of migration [27, 28]. Disadvantageous early life exposures (low birth weight, poor nutrition in early life, socioeconomic disadvantage and poverty), genetic predisposition and exposures to psychosocial stress pre- and post-migration may predispose to a higher incidence of disease, for example T2D, among persons of migrant origin compared with reference European populations [20, 23, 27, 29]. Changes in the environment and the consequent changes in diet, physical activity and other behavioural patterns may contribute to rapid weight gain and hence to changes in glucose levels [30]. Additionally, social and political context within which the person is immersed has significant impact on health outcomes [31].

There is also increasing awareness that there are significant differences in body fat distribution, body shape and size among persons of Middle-Eastern and African origin compared with the general population in European countries, which limits particularly the applicability of abdominal obesity measured with a single measure of WC as a key indicator of the increased risk for T2D among these population groups [16, 18, 21, 32]. It has been previously suggested that the use of continuous anthropometric measures in addition to categorical measures may help disentangle to which extent possible differences across population groups are attributable to methodological issues related to the lack of appropriate obesity cut-off values as opposed to actual differences in the mechanisms leading to T2D [16].

In order to reduce the high societal and economic burden of T2D in the population as a whole, emphasis should be placed on accurate identification of persons with increased risk for T2D taking into account diverse population groups [1]. Furthermore, from the preventive perspective, examining possible variations in the association between obesity and glucose levels before T2D has emerged is of particular value. There is however, scarcity of such data among persons of migrant origin. Availability of biological data is generally limited in studies exploring migrant health and availability of HbA1c measurement is even more restricted. No such studies examining the association between anthropometric measures and glucose levels (fasting blood glucose and/or HbA1c) were found concerning persons of Russian, Somali or Kurdish origin. The aim of this study was to examine whether there are differences in the degree of the association between established anthropometric measures (body mass index (BMI), waist circumference (WC), waist-to-height ratio (WHtR) and waist-to-hip ratio (WHR) and glucose levels (fasting blood glucose and HbA1c) among persons without T2D of Russian, Somali and Kurdish origin in comparison with the general Finnish population. We hypothesised that there are differences in the association between anthropometric measures and glucose levels measured with fasting blood glucose and HbA1c particularly among persons of Somali and Kurdish origin in comparison with the general population in Finland.

## Methods

### Design and study population

This study is based on cross-sectional data of the Migrant Health and Wellbeing Study (Maamu). The study is described in more details elsewhere [33]. Briefly, a stratified random sample of 3000 persons of Russian, Somali and Kurdish origin was drawn from the National Population Register that contains information on all persons with a permanent residence permit in Finland. Inclusion criteria were age 18–64 years, country of birth (Russia/Former Soviet Union, Somalia and Iran/Iraq), mother tongue (Russian/Finnish and Sorani dialect of Kurdish) and minimum one year of residence in Finland. The study was conducted between 2010 and 2012 in six cities (Helsinki, Espoo, Vantaa, Turku, Tampere Vaasa). These six cities were selected for the Maamu Study as they hosted a substantial part of the studied population groups (93% of Somali origin, 67% of Kurdish origin and 47% of Russian origin) at the planning stage of the Maamu Study in 2008. Currently, persons born outside of Finland constitute 7% of the total population [34]. Persons born in Russia or the Former Soviet Union are the largest migrant origin group in Finland. Persons born in Iraq or Iran are the fourth largest population group and the largest migrant origin population group with refugee background. Persons born in Somalia are the fifth largest migrant origin group. [34] The Maamu Study consisted of a structured face-to-face interview and a standardized health examination.

The reference group consists of participants in the Health 2011 Survey representing the general Finnish population. Health 2011 Survey is a population-based health examination survey based on a national stratified random sample drawn from the National Population Register [35]. The current study uses a subset of all of the Health 2011 Survey participants residing in the corresponding cities as the participants of the Maamu Study. Data collection in the Health 2011 Survey followed a comparative protocol to the Maamu Study and consisted of a structured face-to-face interview, standardized health examination and self-completed questionnaires.

The current study is limited to 30–64 year-old health examination participants of the Maamu Study and the Health 2011 Survey. Maamu Study and Health 2011 Survey data were supplemented with register-based data from the Social Institution of Finland on reimbursement rights for diabetes medication and the Finnish Care Register for Health Care on inpatient and outpatient hospital care for diabetes [36]. The Social Institution of Finland data was available for the years between 1998 and 2011 and the Finnish Care Register for Health Care data was available for the years between 1994 and 2012.

Participation rate in the health examination was 48% for Russian, 40% for Somali, 59% for Kurdish origin migrants and 56% for participants in the Health 2011 Survey. Persons with type 1 and type 2 diabetes were excluded from this study (*n* = 146) as their medication affects glucose levels. Type 1 or type 2 diabetes were identified based on having at least one of the following: special reimbursement rights for diabetes medication, diabetes (International Classification of Disease (ICD-10) codes E10 or E11) as reason for healthcare visit/care, self-reported diabetes diagnosis made by a physician, self-reported use of diabetes medications, HbA1c ≥6.5% (48 mmol/mol) or fasting glucose of ≥7.0 mmol/l based on biochemical analysis of blood included in the health examination. Persons without T2D, for whom all the variables used in this study were intact without missing data, and whose glucose fasting was within the 4-17 h fasting range, were included into the study. Therefore, the study is based on the data of 293 Russian origin, 184 Somali origin and 275 Kurdish origin participants in the Maamu Study and 653 participants in the Health 2011 Survey. Mean fasting duration was 13 h for persons of Russian origin, 12 h for persons of Somali and Kurdish origin and 9 h for the general population.

### Clinical measurements and interview data

The health examination in both surveys included measurements of weight, height, waist and hip circumferences according to the European Health Examination Survey standards [37]. Weight was measured wearing light clothing and no shoes with a balanced beam scale (Seca 709) in the Maamu Survey and as a part of the bioimpedance body composition analysis (Seca 514) in the Health 2011 Survey. In both studies, height was measured without shoes with a stand-alone stadiometer (Seca 231). Waist and hip circumference were measured with a non-elastic soft measurement tape to the nearest millimeter. WC was measured half-way between the lowest rib and the top of iliac crest on bare skin or wearing light clothing. Weight and WC were not measured if the participant was over 20 weeks pregnant. Hip circumference was not measured in the Health 2011 Survey and is available for Maamu Survey participants only. Hip circumference was measured at the widest circumference of the buttock over underwear or light clothing, with the participant standing upright, legs close together.

Blood samples were taken by trained laboratory staff, centrifuged within an hour and frozen at − 20 °C on site. Samples were shipped packed in dry ice weekly in Helsinki, Espoo, and Vantaa, and monthly in other cities, to their final storage location at the National Institute for Health and Welfare, where they were stored at − 70 °C. HbA1c was measured with the immuno-turbidimetric method using Abbott Architect reagents. The inter-assay coefficients of variation for HbA1c was 3.9%. Glucose was measured with fluoride citrate plasma in the Maamu Study and serum in the Health 2011 Survey, with inter-essay coefficients of variation of 1.9 and 1.6% respectively. The laboratory (Disease Risk Unit at the National Institute for Health and Welfare) conducting the analyses took part in the External Quality Assessment Schemes organised by Labquality, Helsinki, Finland.

### Variable definition

Age was used as a binary variable (30–44 years vs. 45–64 years) [38]. Both continuous and categorical anthropometric measures were included into the analyses. BMI, WC and WHR were categorized according to the World Health Organization categories of obesity [38]. BMI was calculated as kg/m^2^, with values 25–29.9 kg/m^2^ for overweight and ≥ 30 kg/m^2^ for obesity. WHR was calculated as waist circumference in cm divided by hip circumference in cm. Values of ≥0.90 for men and ≥ 0.85 for women were regarded as obesity [38]. For WC, overweight was categorized as 94–102 cm for men and 80–88 cm for women and obesity as WC > 102 cm for men and > 88 cm for women. WHtR was calculated as waist circumference in cm divided by height in cm [38]. WHtR was categorized as 0.50–0.59 for overweight and ≥ 0.60 for obesity [14].

### Statistical analysis

All analyses accounted for stratified sampling and finite population correction [39]. Sudaan 11.0.1 and SAS 9.3 software packages were used for conducting the analyses [40]. Effects of non-response and different sampling probabilities were accounted for by using inverse probability weights [41] that were calculated for the total sample (both participants and non-respondents) based on age, sex, country of birth, city of residence and marital status using data available from the National Population register. All analyses were adjusted for age. Analyses including fasting glucose were also adjusted for fasting time. Analyses were conducted using categorical and continuous anthropometric measures to reduce methodological bias that the use of inappropriate obesity categorisations may have on the findings. Glucose levels were measured with HbA1c and fasting blood glucose, which are both recommended indicators for blood glucose levels [5, 6].

**Fig. 1:**
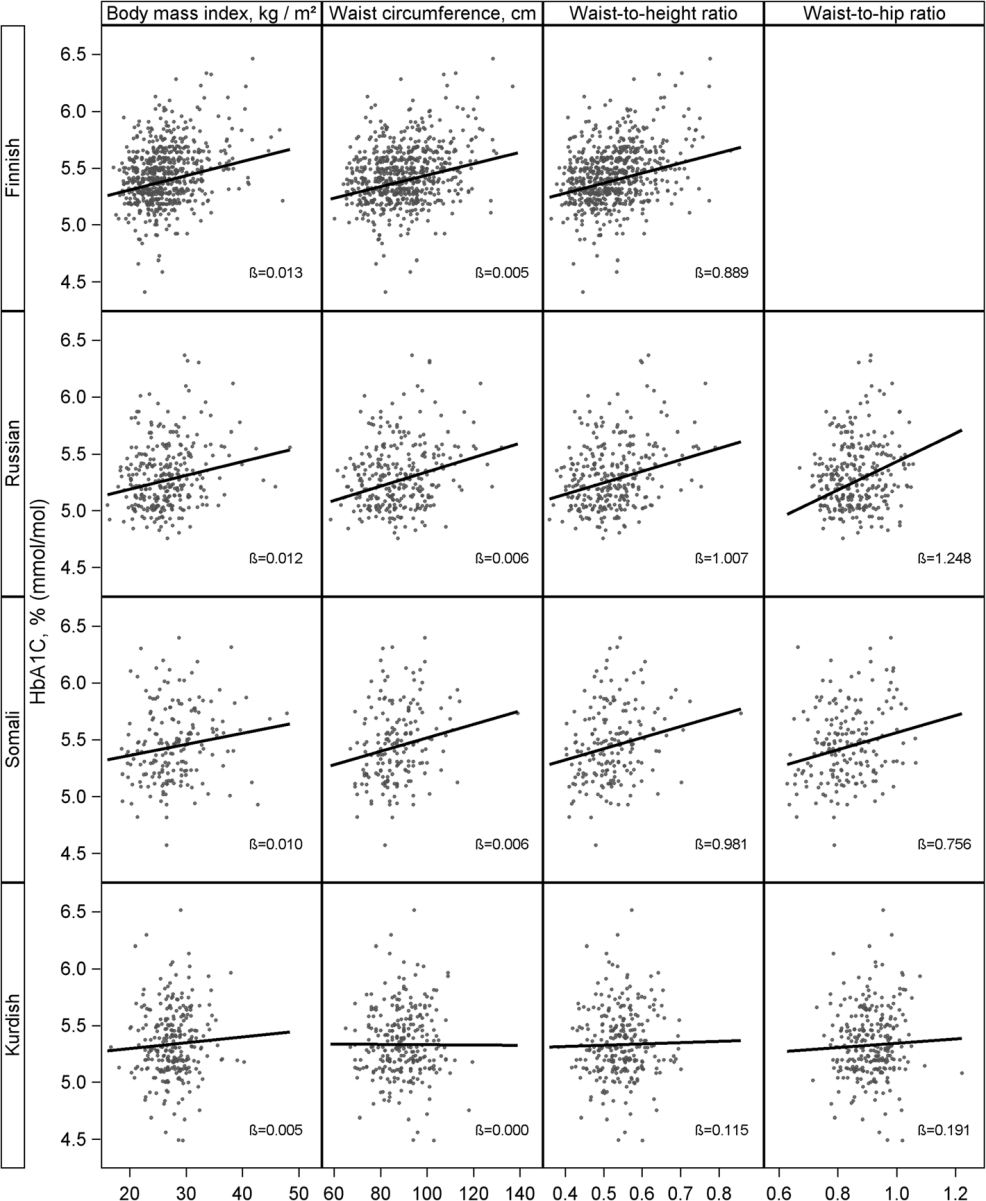
Association between anthropometric measures and glycated haemoglobin (HbA1c)

Linear regression was applied for analysing continuous response variables in Table 1. Statistical significance was assessed with Sattherthwaite F-statistic. Logistic regression was applied for binary and multinomial response variables in Table 2. Mean values in Table 1 and categorical proportion values in Table 2 as well as their 95% confidence intervals were calculated using predictive margins [24]. Linear regression was applied to calculate age and sex-adjusted ß-coefficients for the association between continuous anthropometric measures and HbA1c presented in Fig. 1 and Additional file 1: Figure S1 and Additional file 2: Figure S2. Coefficients of determination (R^2^) presented in Tables 3 and 4 were calculated as an indicator to which extent the dependent variable (HbA1c) is predictable from the independent variables (age, sex, anthropometric measures).

**Table 1:** Age-adjusted descriptive characteristics of persons without diabetes

	General population	Russian origin	Somali origin	Kurdish origin
	mean (95% CI)	mean (95% CI)	mean (95% CI)	mean (95% CI)
Men, N	277	101	67	141
Age, years	46.4 (44.8–48.0)	43.4 (41.3–45.5)^*^	39.6 (37.5–41.7)^***^	40.9 (39.7–42.1)^***^
Weight, kg	85.3 (83.2–87.4)	82.6 (79.6–85.6)	74.0 (71.5–76.5)^***^	80.6 (78.8–82.4)^**^
Height, cm	180.2 (179.1–181.3)	176.7 (175.4–177.9)^***^	175.0 (173.4–176.6)^***^	171.5 (170.5–172.4)^***^
Hip circumference, cm	N/A	100.3 (98.7–101.8)	99.8 (98.3–101.4)	97.1 (96.0–98.2)
Waist circumference	93.9 (92.4–95.4)	92.3 (89.7–94.9)	85.4 (83.5–87.2)^***^	92.8 (91.5–134.8)
Body mass index, kg/m^2^	26.2 (25.7–26.7)	26.5 (25.6–27.4)	24.2 (23.4–25.0)^***^	27.4 (26.9–27.9)^**^
Waist-to-height ratio	0.52 (0.51–0.53)	0.52 (0.51–0.54)	0.49 (0.48–0.50)^***^	0.54 (0.53–0.55)^**^
Waist-to-hip ratio	N/A	0.92 (0.90–0.93)	0.85 (0.84–0.87)	0.95 (0.94–0.96)
HbA1c, mmol/mol	35.2 (34.8–35.6)	33.5 (32.9–34.1)^***^	35.4 (34.7–36.2)	34.8 (34.3–35.4)
HbA1c, %	5.4 (5.3–5.4)	5.2 (5.2–5.3)^***^	5.4 (5.3–5.5)	5.3 (5.3–5.4)
Fasting glucose^a^	5.2 (5.1–5.3)	5.6 (5.5–5.7)^***^	5.6 (5.5–5.7)^***^	5.7 (5.7–5.8)^***^
Women, N	376	192	117	134
Age, years	47.0 (45.9–48.1)	46.7 (45.3–48.0)	41.8 (40.2–43.5)^***^	40.5 (39.4–41.6)^***^
Weight, kg	71.8 (70.3–73.4)	69.9 (68.1–71.8)	79.6 (76.9–82.3)^***^	69.8 (68.2–71.4)
Height	166.1 (165.3–166.9)	163.8 (162.9–164.7)^***^	161.9 (160.8–162.9)^***^	157.5 (156.6–158.3)^***^
Hip circumference, cm	N/A	101.9 (100.4–103.3)	109.4 (107.5–111.3)	99.3 (98.0–100.7)
Waist circumference	86.1 (84.6–87.6)	84.1 (82.3–85.9)	87.5 (85.5–89.5)	86.8 (85.3–88.4)
Body mass index, kg/m^2^	26.1 (25.5–26.6)	26.1 (25.4–26.9)	30.3 (29.4–31.3)^***^	28.1 (27.5–28.7)^***^
Waist-to-height ratio	0.52 (0.51–0.53)	0.51 (0.50–0.53)	0.54 (0.53–0.55)^**^	0.55 (0.54–0.56)^***^
Waist-to-hip ratio	N/A	0.82 (0.81–0.83)	0.80 (0.78–0.82)	0.87 (0.86–0.88)
HbA1c, mmol/mol	35.5 (35.2–35.8)	34.5 (34.0–34.9)^***^	36.2 (35.5–36.9)^*^	34.8 (34.3–35.2)^*^
HbA1c, %	5.4 (5.4–5.4)	5.3 (5.3–5.3)^***^	5.5 (5.4–5.5)^*^	5.4 (5.3–5.4)^*^
Fasting glucose^a^	4.9 (4.9–5.0)	5.5 (5.5–5.6)	5.5 (5.4–5.6)^***^	5.5 (5.4–5.6)^***^

*95% CI* 95% Confidence Interval

*P*-value is presented for the difference between each migrant group and the general Finnish population. ^*^p < 0.05; ^**^p < 0.01; ^***^p < 0.001

^a^Fasting glucose is limited to a 4-17 h fasting range; analyses including glucose are adjusted for fasting

**Table 2:** Prevalence of overweight/obesity with different athropometric indicators in the studied population

	General population % (95% CI)	Russian origin % (95% CI)	Somali origin % (95% CI)	Kurdish origin % (95% CI)
N	653	293	184	275
Age ≥ 45 years, %	56.1 (51.6–60.6)	54.7 (48.5–60.7)	30.8 (24.3–38.2)	30.3 (25.5–35.4)
Body mass index (kg/m^2^)				
< 25	45.3 (40.8–49.9)	42.2 (36.2–48.4)	29.2 (22.7–36.8)	20.5 (16.4–25.2)
25–29.99	37.9 (33.5–42.4)	39.9 (34.1–46.0)	42.4 (35.0–50.2)	55.2 (49.6–60.6)
≥ 30	16.8 (13.6–20.7)	17.9 (13.7–23.1)	28.4 (22.5–35.1)	24.4 (19.9–29.5)
Waist circumference, cm				
< 94 (men)/< 80 (women)	43.5 (39.0–48.0)	45.7 (39.8–51.8)	47.8 (41.2–54.5)	36.1 (31.2–41.3)
94–102 (men)/80–88 (women)	27.2 (23.3–31.5)	28.9 (23.7–34.8)	26.9 (21.0–33.8)	39.4 (34.1–44.9)
> 102 (men)/> 88 (women)	29.3 (25.4–33.6)	25.3 (20.6–30.8)	25.3 (19.8–31.7)	24.6 (20.1–29.7)
Waist-to-height ratio				
< 0.50	42.3 (37.9–46.8)	45.4 (39.4–51.4)	38.7 (31.5–46.4)	22.2 (18.0–26.9)
0.50–0.59	44.0 (39.5–48.7)	41.3 (35.5–47.4)	50.8 (43.0–58.5)	60.0 (54.5–65.3)
≥ 0.60	13.7 (11.1–16.7)	13.3 (9.7–17.9)	10.5 (6.7–16.1)	17.8 (13.9–22.6)
Waist-to-hip ratio				
≥ 0.90 (men)/≥0.85 (women)	N/A	41.0 (35.2–47.0)	27.5 (21.1–34.9)	73.5 (68.5–77.9)

*95% CI* 95% Confidence Interval

**Table 3:** Coefficients of determination for the association between HbA1c and fasting glucose with continuous anthropometric measures

	General population (*n* = 653)		Russian origin (*n* = 293)		Somali origin (*n* = 184)		Kurdish origin (*n* = 275)	
	R^2^ HbA1c	R^2^ FG	R^2^ HbA1c	R^2^ FG	R^2^ HbA1c	R^2^ FG	R^2^ HbA1c	R^2^ FG
Age + sex	0.0660	0.1174	0.1003	0.1027	0.0387	0.0972	0.0079	0.1237
Age + sex + BMI + WC + WHtR + WHR	N/A^a^	N/A^a^	0.1769	0.1936	0.0860	0.2022	0.0335	0.1657
Age + sex + BMI + WC + WHtR	0.1321	0.1683	0.1755	0.1631	0.0732	0.1975	0.0247	0.1652
Age + sex + BMI + WC	0.1244	0.1670	0.1705	0.1495	0.0707	0.1873	0.0195	0.1639
Age + sex + BMI	0.1164	0.1642	0.1340	0.1280	0.0555	0.1510	0.0118	0.1618
Age + sex + WC	0.1243	0.1651	0.1606	0.1447	0.0705	0.1873	0.0079	0.1590
Age + sex + WHtR	0.1314	0.1675	0.1597	0.1503	0.0727	0.1633	0.0088	0.1606
Age + sex + WHR	N/A^a^	N/A^a^	0.1639	0.1894	0.0768	0.1470	0.0094	0.1313

R^2^, coefficient of determination; *HbA1c* glycated haemoglobin; *FG* fasting glucose

*BMI* body mass index, *WC* waist circumference, *WtHR* waist-to-height ratio, *WHR* waist-to-hip ratio

^a^could not be calculated because hip circumference was not measured in the Health 2011 Survey

**Table 4:** Coefficients of determination for the association between HbA1c and fasting glucose with categorical anthropometric measures

	General population (*n* = 653)		Russian origin (*n* = 293)		Somali origin (*n* = 184)		Kurdish origin (*n* = 275)	
	R^2^ HbA1c	R^2^ FG	R^2^ HbA1c	R^2^ FG	R^2^ HbA1c	R^2^ FG	R^2^ HbA1c	R^2^ FG
Age + sex	0.0660	0.1174	0.1003	0.1027	0.0387	0.0972	0.0079	0.1237
Age + sex + BMI + WC + WHtR + WHR	N/A^a^	N/A^a^	0.1778	0.2211	0.0976	0.1970	0.0325	0.1716
Age + sex + BMI + WC + WHtR	0.1196	0.1618	0.1637	0.1901	0.0974	0.1956	0.0283	0.1715
Age + sex + BMI + WC	0.1168	0.1580	0.1575	0.1486	0.0706	0.1705	0.0271	0.1709
Age + sex + BMI	0.1053	0.1543	0.1401	0.1434	0.0559	0.1659	0.0206	0.1672
Age + sex + WC	0.1129	0.1493	0.1510	0.1208	0.0627	0.1341	0.0080	0.1572
Age + sex + WHtR	0.1123	0.1554	0.1509	0.1655	0.0802	0.1758	0.0095	0.1539
Age + sex + WHR	N/A^a^	N/A^a^	0.1470	0.1715	0.0482	0.1317	0.0111	0.1314

R^2^, coefficient of determination; *HbA1c* glycated haemoglobin, *FG* fasting glucose;

*BMI* body mass index, *WC* waist circumference, *WtHR* waist-to-height ratio, *WHR* waist-to-hip ratio

^a^could not be calculated because hip circumference was not measured in the Health 2011 Survey

## Results

Age-adjusted characteristics of the study participants are presented in Table 1. All migrant origin men and women were on average shorter compared with men and women in the general population. Somali origin men had significantly lower weight, WC, BMI and WHtR compared with men in the general population. Somali origin women, in contrast, had significantly higher weight, BMI, WHtR but not WC compared with women in the general population. In addition to being on average approximately 8 cm shorter, Kurdish origin men had lower weight and WC but higher BMI and similar WHtR compared with the reference group. Kurdish origin women had lower weight and similar WC, while BMI and WHtR were significantly higher compared with women in the general population. WHR was not available for the general population. Compared with Russian origin men, for whom anthropometric characteristics were otherwise rather similar as those of the general population, WHR was significantly lower among Somali origin men and higher among Kurdish origin men. WHR was similar among Somali origin and higher among Kurdish origin women compared with Russian origin women. Mean HbA1c values were significantly lower among Russian origin men and women and Kurdish origin women, whereas HbA1c was significantly higher among Somali origin women compared with the general population. Mean fasting glucose was higher among persons of migrant origin compared with the general population.

Interaction between sex, age and each of the anthropometric measures in relation to HbA1c were tested for each study group separately using the models: Model 1 HbA1c = sex + anthropometric measure + age + sex*anthropometric measure, Model 2 HbA1c = sex + anthropometric measure + age + age*anthropometric measure and Model 3 (men and women separately) HbA1c = anthropometric measure + age + age*anthropometric measure. No statistically significant interactions were found in Model 1 and Model 2. In Model 3, a positive interaction between age and each of the anthropometric measures in relation to HbA1c was found among Russian origin women (*p* < 0.05) and for WC, WHtR and WHR in relation to HbA1c among Somali origin men (*p* < 0.05). Further analyses showed that there was no significant association between HbA1c and anthropometric measures in the younger age group (30–44 years) among Russian origin women. However, in the older age group (45–64 years), the associations were significant for all anthropometric measures. Among Somali origin men, the effect was reversed, with significant or almost significant associations found in the younger age group only.

Interactions between sex, age and each of the anthropometric measures in relation to fasting blood glucose were also tested for each study group using the same models. No interaction was found for Model 1 in any of the study groups. A positive interaction was found in Model 2 among the general population for BMI and WC (*p* < 0.05). In Model 3, a positive interaction was found for WC and WHR among Somali origin men (*p* < 0.05), for BMI and WC among Kurdish origin men (*p* < 0.05) and for WC among women in the general population (*p* < 0.05). Upon further analyses, the associations between anthropometric measures and fasting blood glucose were stronger for the older age group (45–64 years) among Kurdish origin men and women in the general population. For Somali origin men, however, the association was stronger in the younger age group and no significant association between anthropometric measures and fasting blood glucose were found in the older age group.

While statistically significant interactions were found among Russian origin women and Somali origin men, no direct conclusions can be made on how these may influence the findings because the size of the stratified population by sex and by study group was relatively small. Furthermore, since the overall association was similar across all study groups, it was feasible to present results for men and women jointly in the rest of the analyses, with age and sex as confounding variables.

Distribution of overweight and obesity among the study participants according to different anthropometric measures is presented in Table 2. Prevalence of overweight and obesity was highest among persons of Kurdish origin. The proportion of those belonging to the normal weight category was similar across BMI, WHtR and WHR among persons of Russian origin and the general population. Higher variation in the proportion of those identified as overweight and obese across different anthropometric measures was observed among persons of Somali and Kurdish origin. Among persons of Somali origin, prevalence of overweight and obesity was highest for BMI and lowest for WC. Among persons of Kurdish origin, prevalence of overweight and obesity was highest for BMI and WHtR and lowest for WC. Abdominal obesity determined by WHR was high among persons of Kurdish origin and low among persons of Somali origin.

Strength of the association between continuous anthropometric measures and HbA1c was relatively similar among persons of Russian, Somali and Kurdish origin (Fig. 1). Compared with the general Finnish population, the difference in the association between continuous anthropometric measures and HbA1c was statistically significant among the Kurdish origin population for WC (*p* = 0.044) and WHtR (*p* = 0.040). The difference was statically significant for WC only (*p* = 0.031) when comparing these two groups using categorical anthropometric measures. No statistically significant differences in the association between continuous or categorical anthropometric measures and fasting blood glucose were found across any of the studied groups (results not shown).

In general, similar observations were made when analyses were performed for men and women separately (Additional file 1: Figure S1 and Additional file 2: Figure S2) as when performed for men and women jointly. An exceptional finding was a high degree of correlation between BMI and HbA1c among Kurdish origin women, which was of a similar strength as among women in the general Finnish population study group. Among Kurdish origin men, however, the strength of the association was low.

Age and sex explained variations in fasting blood glucose to a similar extent among all the study groups (10–12%), whereas variation to which extent age and sex explained variations in HbA1c ranged from < 1% among the Kurdish origin to 10% among the Russian and Somali origin populations (Table 3). Anthropometric measures contribute to explaining the differences in variations in HbA1c to a lesser extent among the Somali and especially among the Kurdish origin population.

Coefficients of determination for the association between categorical anthropometric measures and HbA1c as well as fasting glucose are presented in Table 4. In general, the differences in coefficients of determination were not particularly pronounced across continuous and categorical anthropometric measures. However, categorical WC explained the additional variation to sex and age in fasting blood glucose levels to a lesser extent than continuous WC particularly among the Somali origin study group (3% vs. 9% for categorical and continuous WC respectively). Results of the regression analyses used for calculating the coefficients of determination presented in Tables 3 and 4 are presented for selected models in Additional file 3: Tables S1-S4.

## Discussion

Persons of Kurdish origin had a statistically significantly lower strength of the association between anthropometric measures and HbA1c compared with the general Finnish population, whereas no significant differences were observed across the study groups when examining the strength of the association between anthropometric measures and fasting blood glucose. Although anthropometric measures explained a lower degree of variation in HbA1c also among persons of Somali origin compared with the general population, no statistically significant differences were found in the strength of the association. Therefore, a lower degree of variation is more likely to be due to sample size restrictions rather than actual differences in the degree of the association. There was some variation in whether continuous or categorical anthropometric measures performed better in explaining variation in HbA1c and fasting blood glucose depending on the study group and the specific anthropometric measure examined.

This is a first study examining the association between anthropometric measures and glucose levels among persons without T2D of Russian, Somali and Kurdish origin that have migrated to a high-income country. The association is examined from different angles using several established anthropometric indicators, both categorical and continuous, and two widely used measures of glucose levels (fasting blood glucose and HbA1c). This study provides new insights concerning differences in the association between anthropometric measures and HbA1c among the studied population groups, raising a number of important questions that need to be addressed in future research. While fasting blood glucose and HbA1c are commonly used indicators of glucose levels and are recommended for use in determining the presence of T2D, there are contradicting views whether HbA1c is sufficiently reliable for measuring long term glucose levels also among persons without T2D [5, 6]. World Health Organisation [5] calls for caution in the use of HbA1c to determine pre-diabetes, due to inconclusiveness of previous studies on the degree of correlation between fasting blood glucose and HbA1c [7, 8]. The American Diabetes Association [6], on the other hand, has included HbA1c as an alternative measure for determining pre-diabetes to fasting blood glucose or oral glucose tolerance test.

Genetic factors and disturbances in protein metabolism may cause a higher degree of discordance between fasting blood glucose and HbA1c [7]. HbA1c levels may be influenced by red cell functioning abnormalities, haemoglobinopathies, glycation disturbances, and laboratory assays, which may either produce increased or decreased HbA1c values [5]. In this study, HbA1c was measured with the immuno-turbidimetric method, which in the presence of haemoglobinopathies may produce lower HbA1c values [42].

Haemoglobinopathies are common among some African [43] and Middle-Eastern [44] origin populations.

The extent to which haemoglobinopathies or other possibly co-occuring conditions influence the findings cannot be ascertained in the current study. Further studies should confirm the findings on the low degree of association between anthropometric measures and HbA1c particularly among persons of Kurdish origin. Further studies should also explore the observed association between BMI and HbA1c among Kurdish origin women. Until further studies shed more light on the subject, findings of this study support the cautiousness of WHO experts [5] and hence raise concerns concerning the ADA [6] recommendations towards the use of HbA1c as an interchangeable measure of glucose levels among persons without T2D particularly among persons of Kurdish origin. Since there may be significant variations among population groups originating from the same region, caution should be applied in extending the findings of this study concerning persons of Kurdish origin more broadly to persons of Middle-Eastern origin.

The proportion of persons identified as normal weight were similar among the general Finnish population and the Russian origin study groups. Differences in the sensitivity of anthropometric measures to identify persons with overweight and general or abdominal obesity were observed across different anthropometric measures in the Somali and Kurdish origin study groups. A significantly higher proportion of persons of Somali and Kurdish origin were identified as normal weight using a single WC measurement compared with measures of BMI and WHtR. This may reflect challenges related to the appropriateness of cut-offs established among predominantly White European populations also discussed in previous studies [24, 45, 46]. Additionally, this finding may also be interpreted as support for the use of anthropometric measures that take into account intra-individual and ethnic differences in height and body composition [14].

Considering concerns related to applicability of categorical anthropometric measures established among predominantly White European populations for assessment of overweight and obesity among persons of African and Middle-Eastern origin, it may be feasible to use continuous anthropometric measures in addition to categorical ones when examining the prevalence and risk factors for obesity among populations of diverse origin. Ultimately, however, efforts should be put into setting up prospective cohort studies also among migrant origin populations that will produce data enabling identification of appropriate cut-offs for overweight and obesity also among persons of African and Middle-Eastern origin.

Accumulation of adverse exposures over the life course, including events prior and leading to migration, the migration process itself as well as post-migration exposures may all influence pathways to health among migrating populations [27]. While the length of residence in the country of migration is often used as a proxy for the level of acculturation, it may be that the influence of individual exposures plays a more significant role in health outcomes than length of residence per se [27, 31]. The average length of residence in Finland among the persons included in this study was 11.7 years (95% Confidence Interval (CI) 10.9–12.4) for persons of Russian origin, 13.6 years (95% CI 12.6–14.6) among persons of Somali origin and 11.8 years (11.2–12.3) among persons of Kurdish origin. In our preliminary analyses (not shown), additional adjustment for length of residence did not affect the findings of this study. Although the association between education and health outcomes is well established among the general population in high income countries [47, 48], the role of education appears to be less consistent among persons of migrant origin [26, 49]. In our preliminary analyses (results not shown), adjustment for education also did not impact the findings. Further studies should also address the influence of the complex interaction of migration-related experiences on the observed findings. Migration-related experiences may alter biological mechanisms influencing the onset of risk factors and incidence of chronic disease [27, 28].

### Strengths and limitations

Significant strengths of this study include randomised sampling and inclusion of several diverse population groups into the analyses. Availability of self-report data, biological samples and register-based data allowed for a comprehensive identification of persons with T2D. Further strengths include availability of HbA1c, fasting blood glucose and several objectively measured standardised anthropometric measurements. The use of continuous anthropometric measures reduces bias created by the lack of evidence-based cut-offs for persons of African and Middle-Eastern origin.

This study also has some limitations. The relatively small sample size especially among the Somali origin participants in our study reduces the power of the analyses leading to widened confidence intervals and reduced statistical significance of the findings. Our findings need to be confirmed in a larger sample. Fasting glucose was measured with fluoride citrate plasma among the migrant origin population and with serum among the general Finnish population. While standardization procedures have been taken to reduce the measurement bias, serum samples tend to produce slightly lower glucose values than plasma glucose [50]. Following internal analysis conducted at the National Institute for Health and Welfare, plasma glucose produced approximately 8% higher values than serum (data not published). Therefore, higher fasting glucose values observed among the Maamu Study compared with Health 2011 Survey participants may be influenced by differences in methodologies. A positive interaction between age and anthropometric measures in relation to HbA1c as well as fasting blood glucose was found in some of the study groups. Whether this finding is attributable to sample size restrictions or to actual differences by age group in some of the study populations should be confirmed in a larger sample.

There is a minor possibility of misclassification of participants of this study as belonging to a certain group as the Health 2011 Survey data did not include information on the country of birth, only mother tongue. Mother tongue alone is an insufficiently reliable determinant of first-generation migrants who were included into the sample of the Maamu Study. However, the proportion of those speaking other languages than Finnish or Swedish, which are the two official languages in Finland, was very low among the Health 2011 Survey participants included into this study (< 2%). Out of altogether 11 who spoke a language other than Finnish or Swedish, two persons spoke Russian, none spoke Somali and one spoke Kurdish as their mother tongue. Therefore, it is highly unlikely that this had significant influence on the findings concerning comparison of migrant origin population of the Maamu Study with the general Finnish population of the Health 2011 Survey.

## Conclusions

In conclusion, we found a low degree of association between age, sex and anthropometric measures and HbA1c among persons of Kurdish origin. In contrast, a similar degree of association was observed for age, sex and fasting blood glucose in this study group as in the general population. Based on this observation, use of HbA1c as an indicator of glucose levels appears to be limited among persons of Kurdish origin in the current study. Further studies should explore the underlying mechanisms for this observation. Possible sex differences in the degree of the association between anthropometric measures and glucose indices should also be explored. The degree of the association between anthropometrics and HbA1c also needs to be explored in more detail among persons of Somali origin.

## Additional files

Additional file 1: **Figure S1.** Association between anthropometric measures and glycated haemoglobin (HbA1c) among men. The association between anthropometric measures (body mass index, waist-to-height ratio, waist-to-hip ratio and waist circumference) among men of Russian, Somali and Kurdish origin and the general Finnish population. (TIFF 14668 kb)

Additional file 2: **Figure S2.** Association between anthropometric measures and glycated haemoglobin (HbA1c) among women. The association between anthropometric measures (body mass index, waist-to-height ratio, waist-to-hip ratio and waist circumference) among women of Russian, Somali and Kurdish origin and the general Finnish population. (TIFF 21117 kb)

Additional file 3: **Table S1.** Regression results for the association between continuous anthropometric measures and fasting blood glucose among the studied populations. Age and sex adjusted regression results for the association between continuous anthropometric measures (body mass index, waist-to-height ratio, waist-to-hip ratio and waist circumference) and fasting blood glucose among persons of Russian, Somali and Kurdish origin and persons belonging to the general Finnish population. **Table S2.** Regression results for the association between continuous anthropometric measures and glycated haemoglobin (HbA1c) among the studied populations. Age and sex adjusted regression results for the association between continuous anthropometric measures (body mass index, waist-to-height ratio, waist-to-hip ratio and waist circumference) and HbA1c among persons of Russian, Somali and Kurdish origin and persons belonging to the general Finnish population. **Table S3.** Regression results for the association between categorical anthropometric measures and fasting blood glucose among the studied populations. Age and sex adjusted regression results for the association between categorical anthropometric measures (body mass index, waist-to-height ratio, waist-to-hip ratio and waist circumference) and fasting blood glucose among persons of Russian, Somali and Kurdish origin and persons belonging to the general Finnish population. **Table S4.** Regression results for the association between categorical anthropometric measures and glycated haemoglobin (HbA1c) among the studied populations. Age and sex adjusted regression results for the association between categorical anthropometric measures (body mass index, waist-to-height ratio, waist-to-hip ratio and waist circumference) and HbA1c among persons of Russian, Somali and Kurdish origin and persons belonging to the general Finnish population. (DOC 251 kb)

## Abbreviations

95% CI: 95% Confidence Interval
BMI: Body mass index
FG: Fasting glucose
HbA1c: Glycated haemoglobin
ICD-10: International Classification of Diseases
Maamu Study: Migrant Health and Wellbeing Study
R^2^: Coefficients of determination
ref. group: Reference group (the general Finnish population)
T2D: Type 2 diabetes
WC: Waist circumference
WHR: Waist-to-hip ratio
WtHR: Waist-to-height ratio
