# Supplementary figures and images for: The association between anthropometric measures and glycated haemoglobin (HbA1c) is different in Russian, Somali and Kurdish origin migrants compared with the general population in Finland: a cross-sectional population-based study
Source: BMC Public Health. 2019 Apr 11;19:391. doi: 10.1186/s12889-019-6698-0 (PMC6458679; doi:10.1186/s12889-019-6698-0)

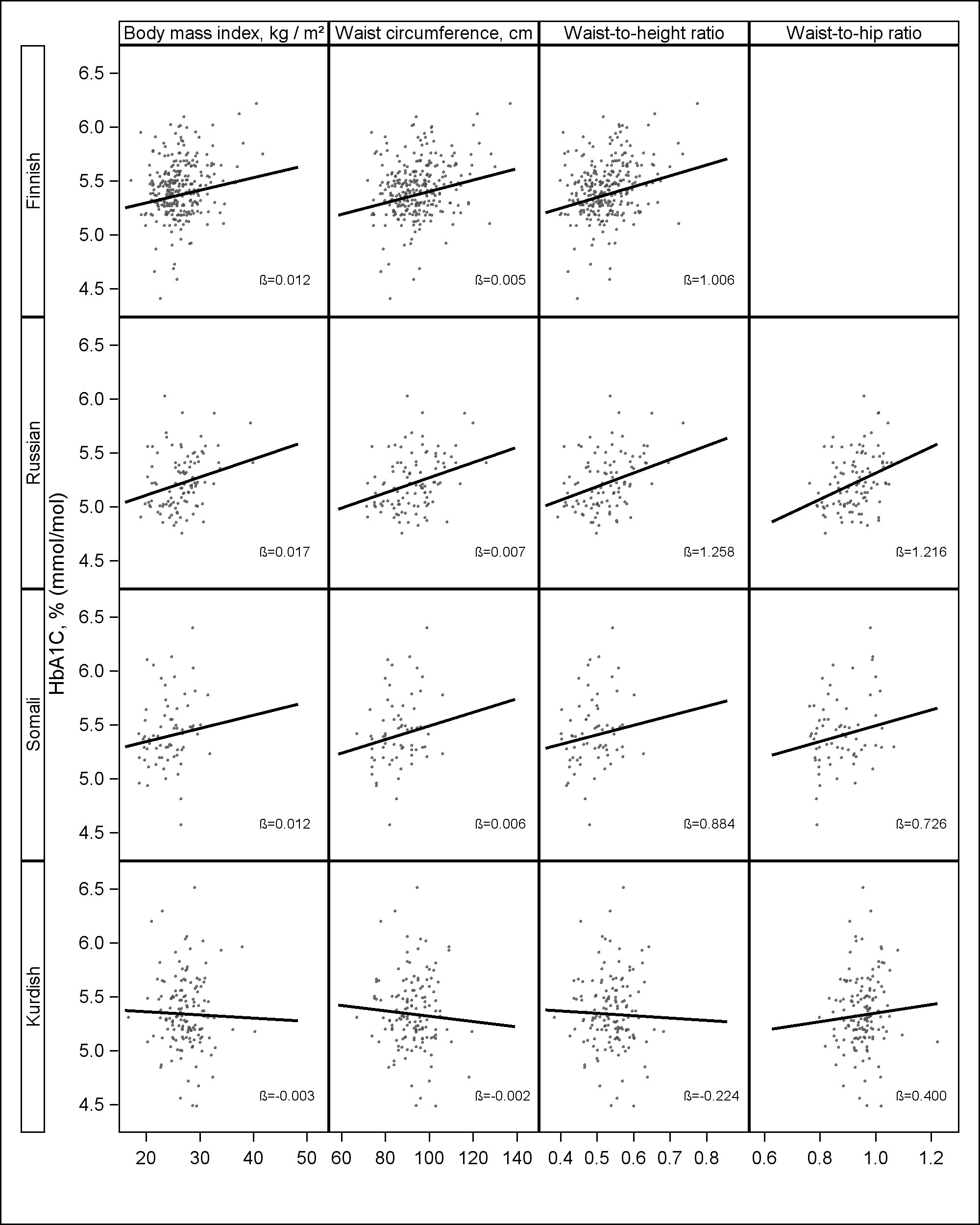

Supplement: Supplementary file 1 — Figure S1. Association between anthropometric measures and glycated haemoglobin (HbA1c) among men. The association between anthropometric measures (body mass index, waist-to-height ratio, waist-to-hip ratio and waist circumference) among men of Russian, Somali and Kurdish origin and the general Finnish population. (TIFF 14668 kb) [file 12889_2019_6698_MOESM1_ESM.tiff]

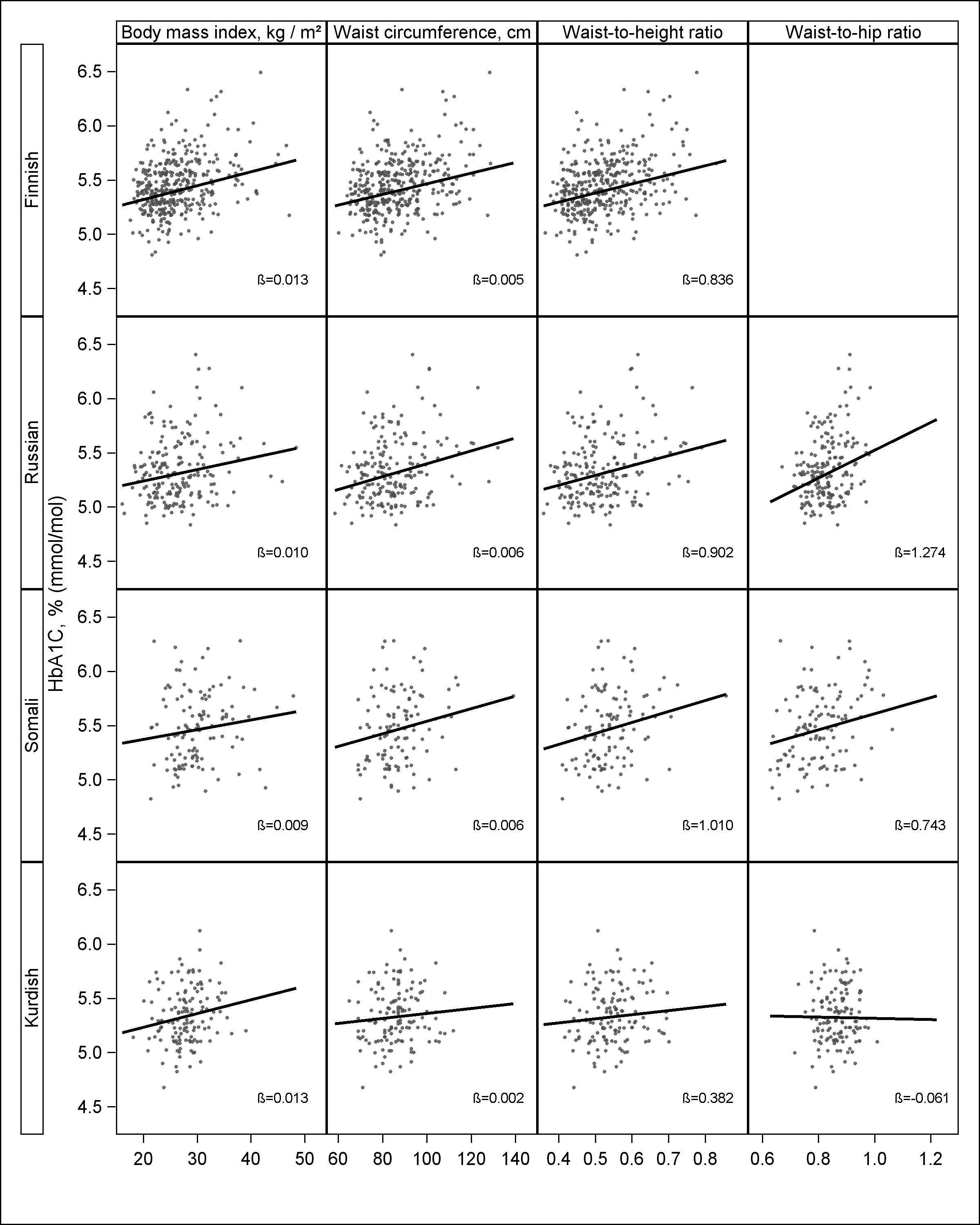

Supplement: Supplementary file 2 — Figure S2. Association between anthropometric measures and glycated haemoglobin (HbA1c) among women. The association between anthropometric measures (body mass index, waist-to-height ratio, waist-to-hip ratio and waist circumference) among women of Russian, Somali and Kurdish origin and the general Finnish population. (TIFF 21117 kb) [file 12889_2019_6698_MOESM2_ESM.tiff]
