# Supplementary material for: The association between anthropometric measures and glycated haemoglobin (HbA1c) is different in Russian, Somali and Kurdish origin migrants compared with the general population in Finland: a cross-sectional population-based study
Source: BMC Public Health. 2019 Apr 11;19:391. doi: 10.1186/s12889-019-6698-0 (PMC6458679; doi:10.1186/s12889-019-6698-0)
Supplement: Supplementary file 3 — Table S1. Regression results for the association between continuous anthropometric measures and fasting blood glucose among the studied populations. Age and sex adjusted regression results for the association between continuous anthropometric measures (body mass index, waist-to-height ratio, waist-to-hip ratio and waist circumference) and fasting blood glucose among persons of Russian, Somali and Kurdish origin and persons belonging to the general Finnish population. Table S2. Regression results for the association between continuous anthropometric measures and glycated haemoglobin (HbA1c) among the studied populations. Age and sex adjusted regression results for the association between continuous anthropometric measures (body mass index, waist-to-height ratio, waist-to-hip ratio and waist circumference) and HbA1c among persons of Russian, Somali and Kurdish origin and persons belonging to the general Finnish population. Table S3. Regression results for the association between categorical anthropometric measures and fasting blood glucose among the studied populations. Age and sex adjusted regression results for the association between categorical anthropometric measures (body mass index, waist-to-height ratio, waist-to-hip ratio and waist circumference) and fasting blood glucose among persons of Russian, Somali and Kurdish origin and persons belonging to the general Finnish population. Table S4. Regression results for the association between categorical anthropometric measures and glycated haemoglobin (HbA1c) among the studied populations. Age and sex adjusted regression results for the association between categorical anthropometric measures (body mass index, waist-to-height ratio, waist-to-hip ratio and waist circumference) and HbA1c among persons of Russian, Somali and Kurdish origin and persons belonging to the general Finnish population. (DOC 251 kb) [file 12889_2019_6698_MOESM3_ESM.doc]

|  | | | General population | | Russian origin | | Somali origin | | Kurdish origin | |
| --- | --- | --- | --- | --- | --- | --- | --- | --- | --- | --- |
|  | | | ß | p-value | ß | p-value | ß | p-value | ß | p-value |
| Age + sex+ BMI | | |  |  |  |  |  |  |  |  |
|  | Intercept | | 4.2966 |  | 5.9708 |  | 5.2143 |  | 5.2565 |  |
|  | Age | |  |  |  |  |  |  |  |  |
|  |  | 30-44 | -0.1985 | <0.0001 | -0.1835 | 0.0004 | -0.2261 | 0.0007 | -0.1314 | 0.0083 |
|  |  | 45-64 | ref. |  | ref. |  | ref. |  | ref. |  |
|  | Sex | |  |  |  |  |  |  |  |  |
|  |  | men | 0.2472 | <0.0001 | 0.1462 | 0.0078 | 0.2694 | 0.0003 | 0.2557 | <0.0001 |
|  |  | women | ref. |  | ref. |  | ref. |  | ref. |  |
|  | BMI | | 0.0240 | <0.0001 | 0.0147 | 0.0041 | 0.0238 | 0.0051 | 0.0257 | 0.0002 |
| Age + sex + WC | | |  |  |  |  |  |  |  |  |
|  | Intercept | | 4.1289 |  | 5.7373 |  | 4.7306 |  | 5.1455 |  |
|  | Age | |  |  |  |  |  |  |  |  |
|  |  | 30-44 | -0.1872 | <0.0001 | -0.1587 | 0.0020 | -0.1800 | 0.0075 | -0.1189 | 0.0177 |
|  |  | 45-64 | ref. |  | ref. |  | ref. |  | ref. |  |
|  | Sex | |  |  |  |  |  |  |  |  |
|  |  | men | 0.1782 | 0.0003 | 0.0878 | 0.1419 | 0.1502 | 0.0186 | 0.1817 | 0.0003 |
|  |  | women | ref. |  | ref. |  | ref. |  | ref. |  |
|  | WC | | 0.0090 | <0.0001 | 0.0075 | 0.0002 | 0.0138 | <0.0001 | 0.0094 | 0.0002 |
| Age + sex + WHtR | | |  |  |  |  |  |  |  |  |
|  | Intercept | | 4.1056 |  | 5.7020 |  | 4.9018 |  | 5.1373 |  |
|  | Age | |  |  |  |  |  |  |  |  |
|  |  | 30-44 | -0.1784 | 0.0001 | -0.1470 | 0.0039 | -0.1887 | 0.0048 | -0.1120 | 0.0263 |
|  |  | 45-64 | ref. |  | ref. |  | ref. |  | ref. |  |
|  | Sex | |  |  |  |  |  |  |  |  |
|  |  | men | 0.2453 | <0.0001 | 0.1369 | 0.0118 | 0.2198 | 0.0012 | 0.2523 | <0.0001 |
|  |  | women | ref. |  | ref. |  | ref. |  | ref. |  |
|  | WHR | | 1.5287 | <0.0001 | 1.2732 | <0.0001 | 1.8792 | 0.0003 | 1.5165 | 0.0002 |
| Age + sex + WHR | | |  |  |  |  |  |  |  |  |
|  | Intercept | | N/A |  | 4.6650 |  | 4.9194 |  | 5.3794 |  |
|  | Age | |  |  |  |  |  |  |  |  |
|  |  | 30-44 | N/A |  | -0.1331 | 0.0060 | -0.1837 | 0.0073 | -0.1349 | 0.0079 |
|  |  | 45-64 |  |  | ref. |  | ref. |  | ref. |  |
|  | Sex | |  |  |  |  |  |  |  |  |
|  |  | men | N/A |  | -0.0411 | 0.5343 | 0.0603 | 0.3882 | 0.1826 | 0.0027 |
|  |  | women |  |  |  |  |  |  |  |  |
|  | WHtR | | N/A |  | 2.0248 | <0.0001 | 1.1894 | 0.0025 | 0.7087 | 0.0651 |

Table S1 Regression results for the association between continuous anthropometric measures and fasting blood glucose among the studied populations

BMI, body mass index; WC, waist circumference; WtHR, waist-to-height ratio; WHR, waist-to-hip ratio.

Table S2 Regression results for the association between continuous anthropometric measures and glycated haemoglobin (HbA1c) among the studied populations

|  | | | General population | | Russian origin | | Somali origin | | Kurdish origin | |
| --- | --- | --- | --- | --- | --- | --- | --- | --- | --- | --- |
|  | | | ß | p-value | ß | p-value | ß | p-value | ß | p-value |
| Age + sex+ BMI | | |  |  |  |  |  |  |  |  |
|  | Intercept | | 5.1281 |  | 5.0670 |  | 5.2302 |  | 5.1944 |  |
|  | Age, years | |  |  |  |  |  |  |  |  |
|  |  | 30-44 | -0.1120 | <0.0001 | -0.1354 | 0.0002 | -0.116 | 0.0383 | -0.0521 | 0.1997 |
|  |  | 45-64 | ref. |  | ref. |  | ref. |  | ref. |  |
|  | Sex | |  |  |  |  |  |  |  |  |
|  |  | men | -0.0230 | 0.2884 | -0.0792 | 0.0330 | -0.0044 | 0.9436 | 0.0211 | 0.5248 |
|  |  | women | ref. |  | ref. |  | ref. |  | ref. |  |
|  | BMI | | 0.0125 | <0.0001 | 0.0116 | 0.0018 | 0.0097 | 0.1509 | 0.0054 | 0.2640 |
| Age + sex + WC | | |  |  |  |  |  |  |  |  |
|  | Intercept | | 5.0211 |  | 4.8483 |  | 4.9926 |  | 5.3368 |  |
|  | Age, years | |  |  |  |  |  |  |  |  |
|  |  | 30-44 | -0.1040 | <0.0001 | -0.1141 | 0.0016 | -0.0917 | 0.0870 | -0.0538 | 0.1853 |
|  |  | 45-64 | ref. |  | ref. |  | ref. |  | ref. |  |
|  | Sex | |  |  |  |  |  |  |  |  |
|  |  | men | -0.0611 | 0.0153 | -0.1274 | 0.0014 | -0.0522 | 0.2862 | 0.0182 | 0.5969 |
|  |  | women | ref. |  | ref. |  | ref. |  | ref. |  |
|  | WC | | 0.0050 | <0.0001 | 0.0061 | <0.0001 | 0.0059 | 0.0133 | 0.0001 | 0.9522 |
| Age + sex + WHtR | | |  |  |  |  |  |  |  |  |
|  | Intercept | | 4.9907 |  | 4.8583 |  | 4.9840 |  | 5.2572 |  |
|  | Age | |  |  |  |  |  |  |  |  |
|  |  | 30-44 | -0.0982 | <0.0001 | -0.1082 | 0.0030 | -0.0911 | 0.0891 | -0.0510 | 0.2118 |
|  |  | 45-64 | ref. |  | ref. |  | ref. |  | ref. |  |
|  | Sex | |  |  |  |  |  |  |  |  |
|  |  | men | -0.0240 | 0.2648 | -0.0864 | 0.0183 | -0.0142 | 0.7854 | 0.0196 | 0.5549 |
|  |  | women | ref. |  | ref. |  | ref. |  | ref. |  |
|  | WHtR | | 0.8766 | <0.0001 | 0.9689 | <0.0001 | 0.9790 | 0.0096 | 0.1595 | 0.5577 |
| Age + sex + WHR | | |  |  |  |  |  |  |  |  |
|  | Intercept | | N/A |  | 4.3832 |  | 4.8981 |  | 5.1654 |  |
|  | Age | |  |  |  |  |  |  |  |  |
|  |  | 30-44 | N/A |  | -0.1119 | 0.0017 | -0.0821 | 0.1257 | -0.0523 | 0.1922 |
|  |  | 45-64 | ref. |  | ref. |  | ref. |  | ref. |  |
|  | Sex | |  |  |  |  |  |  |  |  |
|  |  | men | N/A |  | -0.1874 | <0.0001 | -0.1056 | 0.0351 | 0.0007 | 0.9868 |
|  |  | women | ref. |  | ref. |  | ref. |  | ref. |  |
|  | WHR | | N/A |  | 1.1838 | <0.0001 | 0.7580 | 0.0102 | 0.2075 | 0.4835 |

BMI, body mass index; WC, waist circumference; WtHR, waist-to-height ratio; WHR, waist-to-hip ratio.

Table S3 Regression results for the association between categorical anthropometric measures and fasting blood glucose among the studied populations

|  | | | General population | | Russian origin | | Somali origin | | Kurdish origin | |
| --- | --- | --- | --- | --- | --- | --- | --- | --- | --- | --- |
|  | | | ß | p-value | ß | p-value | ß | p-value | ß | p-value |
| Age + sex+ BMI | | |  |  |  |  |  |  |  |  |
|  | Intercept | | 4.8410 | <0.0001 | 6.2858 | <0.0001 | 5.7467 | <0.0001 | 5.8376 | <0.0001 |
|  | Age, years | |  |  |  |  |  |  |  |  |
|  |  | 30-44 | -0.2044 | <0.0001 | -0.1813 | 0.0005 | 0.2157 | 0.0009 | -0.1424 | 0.0041 |
|  |  | 45-64 | ref. |  | ref. |  | ref. |  | ref. |  |
|  | Sex | |  |  |  |  |  |  |  |  |
|  |  | men | 0.2552 | <0.0001 | 0.1473 | 0.0090 | -0.2850 | 0.0001 | 0.2571 | <0.0001 |
|  |  | women | ref. |  | ref. |  | ref. |  | ref. |  |
|  | BMI, kg/m^2^ | |  |  |  |  |  |  |  |  |
|  |  | ≥ 30 | 0.2771 | 0.0238 | 0.2410 | 0.0005 | 0.3461 | 0.0003 | 0.2873 | <0.0001 |
|  |  | 25-29.99 | 0.0700 | <0.0001 | 0.0766 | 0.1872 | 0.2470 | 0.0009 | 0.1407 | 0.0090 |
|  |  | < 25 | ref. |  | ref. | ref. | ref. |  | ref. |  |
| Age + sex + WC | | |  |  |  |  |  |  |  |  |
|  | Intercept | | 4.6030 | <0.0001 | 6.2990 | <0.0001 | 5.5471 | <0.0001 | 5.8631 | <0.0001 |
|  | Age | |  |  |  |  |  |  |  |  |
|  |  | 30-44 | 0.2038 | <0.0001 | -0.1802 | 0.0008 | 0.1963 | 0.0040 | -0.1363 | 0.0066 |
|  |  | 45-64 | ref. |  | ref. |  | ref. |  | ref. |  |
|  | Sex | |  |  |  |  |  |  |  |  |
|  |  | men | 0.2851 | <0.0001 | 0.1661 | 0.0020 | 0.2629 | 0.0019 | 0.3001 | <0.0001 |
|  |  | women | ref. |  | ref. |  | ref. |  | ref. |  |
|  | WC, cm | |  |  |  |  |  |  |  |  |
|  | >102(m)/  >88 (w) | | 0.2200 | <0.0001 | 0.1367 | 0.0228 | 0.2605 | 0.0099 | 0.2299 | 0.0003 |
|  | 94-102 (m)/  80-88 (w) | | 0.0725 | 0.2388 | 0.0071 | 0.9069 | 0.1490 | 0.0866 | 0.1196 | 0.0295 |
|  | < 94 (m)/  <80 (w) | | ref. |  | ref. |  | ref. |  | ref. |  |
| Age + sex + WHtR | | |  |  |  |  |  |  |  |  |
|  | Intercept | | 4.6528 | <0.0001 | 6.2402 | <0.0001 | 5.8121 | <0.0001 | 5.8679 | <0.0001 |
|  | Age, years | |  |  |  |  |  |  |  |  |
|  |  | 30-44 | 0.1868 | 0.0001 | -0.1531 | 0.0021 | 0.1847 | 0.0045 | -0.1218 | 0.0155 |
|  |  | 45-64 | ref. |  | ref. |  | ref. |  | ref. |  |
|  | Sex | |  |  |  |  |  |  |  |  |
|  |  | men | 0.2645 | <0.0001 | 0.1291 | 0.0149 | -0.2102 | 0.0017 | 0.2515 | <0.0001 |
|  |  | women | ref. |  | ref. |  | ref. |  | ref. |  |
|  | WHtR | |  |  |  |  |  |  |  |  |
|  |  | ≥ 0.60 | 0.3078 | <0.0001 | 0.3151 | <0.0001 | 0.4293 | 0.0006 | 0.2598 | 0.0008 |
|  |  | 0.50-0.59 | 0.0638 | 0.2025 | 0.1515 | 0.0043 | 0.1915 | 0.0048 | 0.1156 | 0.0267 |
|  |  | < 0.50 | ref. |  | ref. |  | ref. |  | ref. |  |
| Age + sex + WHR | | |  |  |  |  |  |  |  |  |
|  | Intercept | | N/A |  | 6.1361 | <0.0001 | 5.6366 | <0.0001 | 5.9306 | <0.0001 |
|  | Age, years | |  |  |  |  |  |  |  |  |
|  |  | 30-44 | N/A |  | 0.1556 | 0.0011 | 0.2082 | 0.0020 | -0.1312 | 0.0104 |
|  |  | 45-64 |  |  | ref. |  | ref. |  |  |  |
|  | Sex | |  |  |  |  |  |  |  |  |
|  |  | men | N/A |  | 0.0778 | 0.1587 | 0.1392 | 0.0347 | 0.2248 | <0.0001 |
|  |  | women |  |  | ref. |  | ref. |  | ref. |  |
|  | WHR | |  |  |  |  |  |  |  |  |
|  |  | ≥ 0.90 (m)/  ≥ 0.80 (w) | N/A |  | 0.2423 | <0.0001 | 0.1891 | 0.0033 | 0.0939 | 0.0572 |
|  |  | < 0.90 (m)/ < 0.80 (w) |  |  | ref. |  | ref. |  | ref. |  |

BMI, body mass index; WC, waist circumference; WtHR, waist-to-height ratio; WHR, waist-to-hip ratio.

Table S4 Regression results for the association between categorical anthropometric measures and glycated haemoglobin (HbA1c) among the studied populations

|  | | | General population | | Russian origin | | Somali origin | | Kurdish origin | |
| --- | --- | --- | --- | --- | --- | --- | --- | --- | --- | --- |
|  | | | ß | p-value | ß | p-value | ß | p-value | ß | p-value |
| Age + sex+ BMI | | |  |  |  |  |  |  |  |  |
|  | Intercept | | 5.4115 | <0.0001 | 5.3385 | <0.0001 | 5.3542 | <0.0001 | 5.3277 | <0.0001 |
|  | Age, years | |  |  |  |  |  |  |  |  |
|  |  | 30-44 | -0.1147 | <0.0001 | -0.1426 | <0.0001 | 0.1112 | 0.0389 | -0.0588 | 0.1444 |
|  |  | 45-64 | ref. |  | ref. |  | ref. |  | ref. |  |
|  | Sex | |  |  |  |  |  |  |  |  |
|  |  | men | -0.0223 | 0.2967 | -0.0713 | 0.0604 | 0.0250 | 0.6856 | 0.0239 | 0.4673 |
|  |  | women | ref. |  | ref. |  | ref. |  | ref. |  |
|  | BMI, kg/m^2^ | |  |  |  |  |  |  |  |  |
|  |  | ≥ 30 | 0.1423 | <0.0001 | 0.1538 | 0.0048 | 0.0868 | 0.2681 | 0.0828 | 0.0871 |
|  |  | 25-29.99 | 0.0527 | 0.0262 | 0.0120 | 0.7547 | -0.02174 | 0.7329 | 0.0009 | 0.9827 |
|  |  | < 25 | ref. |  | ref. |  | ref. |  | ref. |  |
| Age + sex + WC | | |  |  |  |  |  |  |  |  |
|  | Intercept | | 5.2758 | <0.0001 | 5.3100 | <0.0001 | 5.3198 | <0.0001 | 5.3405 | <0.0001 |
|  | Age | |  |  |  |  |  |  |  |  |
|  |  | 30-44 | 0.1117 | <0.0001 | -0.1206 | 0.0013 | 0.0938 | 0.0850 | -0.0533 | 0.1881 |
|  |  | 45-64 | ref. |  | ref. |  | ref. |  | ref. |  |
|  | Sex | |  |  |  |  |  |  |  |  |
|  |  | men | -0.0007 | 0.9714 | -0.0584 | 0.1007 | 0.0186 | 0.7728 | 0.0205 | 0.5663 |
|  |  | women | ref. |  | ref. |  | ref. |  | ref. |  |
|  | WC, cm | |  |  |  |  |  |  |  |  |
|  | >102(m)/  >88 (w) | | 0.1338 | <0.0001 | 0.1581 | 0.0008 | 0.1493 | 0.0451 | 0.0048 | 0.9120 |
|  | 94-102 (m)/  80-88 (w) | | 0.0626 | 0.0111 | 0.0130 | 0.7400 | 0.0990 | 0.1492 | 0.0079 | 0.8493 |
|  | < 94 (m)/  <80 (w) | | ref. |  | ref. |  | ref. |  | ref. |  |
| Age + sex + WHtR | | |  |  |  |  |  |  |  |  |
|  | Intercept | | 5.2897 | <0.0001 | 5.3330 | <0.0001 | 5.3045 | <0.0001 | 5.3248 | <0.0001 |
|  | Age, years | |  |  |  |  |  |  |  |  |
|  |  | 30-44 | 0.1028 | <0.0001 | -0.1345 | 0.0001 | 0.0897 | 0.0870 | -0.0509 | 0.2128 |
|  |  | 45-64 | ref. |  | ref. |  | ref. |  | ref. |  |
|  | Sex | |  |  |  |  |  |  |  |  |
|  |  | men | -0.0231 | 0.2976 | -0.0728 | 0.0482 | 0.0191 | 0.7008 | 0.0194 | 0.5619 |
|  |  | women | ref. |  | ref. |  | ref. |  | ref. |  |
|  | WHtR | |  |  |  |  |  |  |  |  |
|  |  | ≥ 0.60 | 0.1622 | <0.0001 | 0.1944 | 0.0003 | 0.2064 | 0.0230 | 0.0389 | 0.4519 |
|  |  | 0.50-0.59 | 0.0785 | 0.0009 | 0.0191 | 0.6049 | 0.1147 | 0.0251 | 0.0210 | 0.5918 |
|  |  | < 0.50 | ref. |  | ref. |  | ref. |  | ref. |  |
| Age + sex + WHR | | |  |  |  |  |  |  |  |  |
|  | Intercept | | N/A |  | 5.1982 | <0.0001 | 5.3911 | <0.0001 | 5.3181 | <0.0001 |
|  | Age, years | | N/A |  |  |  |  |  |  |  |
|  |  | 30-44 |  |  | 0.1262 | 0.0004 | 0.1048 | 0.0502 | -0.0500 | 0.2095 |
|  |  | 45-64 |  |  | ref. |  | ref. |  | ref. |  |
|  | Sex | | N/A |  |  |  |  |  |  |  |
|  |  | men |  |  | -0.1163 | 0.0031 | -0.0576 | 0.2431 | 0.0103 | 0.7576 |
|  |  | women |  |  | ref. |  | ref. |  | ref. |  |
|  | WHR | | N/A |  |  |  |  |  |  |  |
|  |  | ≥ 0.90 (m)/  ≥ 0.80 (w) |  |  | 0.1361 | 0.0005 | 0.0725 | 0.1874 | 0.0404 | 0.2701 |
|  |  | < 0.90 (m)/ < 0.80 (w) |  |  | ref. |  | ref. |  | ref. |  |

BMI, body mass index; WC, waist circumference; WtHR, waist-to-height ratio; WHR, waist-to-hip ratio.
